# Effectiveness of aripiprazole once-monthly in schizophrenia patients pretreated with oral aripiprazole: a 6-month, real-life non-interventional study

**DOI:** 10.1186/s12888-018-1946-x

**Published:** 2018-11-14

**Authors:** Daniel Schöttle, Wolfgang Janetzky, Daniel Luedecke, Elmar Beck, Christoph U. Correll, Klaus Wiedemann

**Affiliations:** 1 Klinik für Psychiatrie und Psychotherapie, Zentrum für Psychosoziale Medizin, Universitätsklinikum Hamburg-Eppendorf, Martinistrasse 52, 20246 Hamburg, Germany; 2 Lundbeck GmbH, Ericusspitze 2, 20457 Hamburg, Germany; 3 ANFOMED GmbH, Röttenbacher Str. 17, 91096 Möhrendorf, Germany; 4 The Zucker Hillside Hospital, Department of Psychiatry, Northwell Health, 75-59 263rd St, Glen Oaks, NY 11004 USA; 5 Hofstra Northwell School of Medicine, Department of Psychiatry and Molecular Medicine, 500 Hofstra Blvd, Hempstead, NY 11549 USA; 6 Charité Universitätsmedizin, Department of Child and Adolescent Psychiatry, Augustenburger Platz 1 (Mittelallee 5A), 13353 Berlin, Germany

**Keywords:** Long-acting injectable, LAI, Naturalistic, Schizophrenia, Schizoaffective

## Abstract

**Background:**

In this study, the treatment of schizophrenia patients with aripiprazole once-monthly (AOM) was evaluated under real-life conditions in a naturalistic setting.

**Methods:**

This multicenter, prospective, non-interventional study included 242 patients (age = 43.1 ± 15.1 years, 55.0% male) who were monitored during 6 months of AOM treatment. Endpoints included measurements of psychopathology (Brief Psychiatric Rating Scale, BPRS) and severity of illness scales (Clinical Global Impressions-Severity, CGI-S, and -Improvement, CGI-I). Furthermore, treatment-related adverse events (TRAEs) were recorded.

**Results:**

At baseline, the mean BPRS total score was 54.1 ± 15.6, the mean CGI-S was 4.8 ± 0.8 and the most frequent illness category was ‘markedly ill’ (41.7%). Patients had been pretreated with oral aripiprazole for a mean duration of 9.7 months (SD: 22.3) and 87.9% were deemed by their clinician as “clinically stable” and for a mean of 5.9 months. The difference in global BPRS after 6 months was − 13.8 (SD: 16.0; 95% CI: [− 15.9; − 11.7]; *p* < 0.001). The proportion of patients with high CGI-S scores decreased and the proportion of patients with low scores increased significantly (*p* < 0.001, respectively). BPRS scores improved numerically especially well in younger patients ≤35 years, CGI-S scores decreased significantly more in this population. TRAEs were rare, with low incidences of extrapyramidal symptoms (2.9%) or weight increase (0.4%).

**Conclusions:**

Treatment with AOM showed satisfying effectiveness in outpatients with further improvement of psychopathology after oral aripiprazole treatment for a considerable duration and even after having achieved clinically judged “stability”. Our findings indicate a robust therapeutic effect of AOM and substantiate previous results from randomized controlled trials under real-world routine conditions.

## Background

Schizophrenia is one of the most complex psychiatric disorders often leading to considerable disability and affecting the lives of patients and their families profoundly [1, 2]. Non- or partial adherence to psychopharmacological therapy is one of the major risk factors for relapses [3, 4], which severely diminish the psychosocial and occupational functioning of patients and negatively affect their quality of life [3, 5, 6]. Treatment with antipsychotics, as a major component in a framework of social and psychological therapies, can help to overcome these impairments and is highly effective [2]. However, nonadherence to treatment in patients with schizophrenia is frequently observed [4, 7, 8] with a significantly heightened risk of subsequent relapses [9]. Even small gaps in taking medication can have a significant effect, as stopping medication for as little as one to 10 days in a one-year period (partial adherence) was found to be associated with a significantly increased risk of hospitalization, with an odds ratio of 1.98 [10], while intermittent use of medication leads to a 3-fold higher risk of relapse in stable patients [11]. Improving adherence to medication may be achieved by using long-acting injectable antipsychotics (LAIs) which may in turn reduce the risk of relapse and improve patient functioning [12–14].

Aripiprazole once-monthly (AOM) is an atypical LAI with a unique pharmacological profile: aripiprazole shows partial agonist activity at dopamine D2/D3 receptors [15, 16], therefore the risk of adverse effects, such as parkinsonism, hyperprolactinemia and sexual dysfunction [17] is low. Effects on serotonin receptors include a partial agonist activity at the 5-HT_1A_ receptor [18] and antagonist activity at 5-HT_2A_ receptor [19], which adds to the antipsychotic profile. The efficacy and tolerability of AOM in patients with schizophrenia has been demonstrated by two randomized, double-blind, controlled studies (RCTs) conducted in the United States [20] and Europe [21]. In the study by Kane et al., 10% of patients in the active group and 39.6% in the placebo group experienced exacerbation of psychotic symptoms or impending relapse (hazard ratio = 5.03) at study endpoint. Altogether, 2.6% vs. 3.7% of patients were hospitalized because of relapse. In the European study, Kaplan–Meier estimated impending relapse rates at week 26 from randomization were 7.1% for AOM 400 mg, 7.8% for oral aripiprazole (10–30 mg/day) and 21.8% for aripiprazole once-monthly at a subtherapeutic dose (50 mg) [21]. AOM 400 mg has a long half-life of 46.5 days [22], which potentially can provide long-term relapse protection for patients.

In the present non-interventional study, the course of schizophrenia was monitored in patients receiving AOM treatment under usual care conditions. The primary endpoint was the assessment of AOM effectiveness on psychopathology by the attending psychiatrist, using the Brief Psychiatric Rating Scale (BPRS). Secondary endpoints included measurement of illness severity and illness improvement using the Clinical Global Impressions- Severity (CGI-S) and - Improvement (CGI-I) scales and the documentation of treatment related adverse events (TRAE). In addition, other instruments measuring functional status and wellbeing of the patients were applied such as the Global Assessment of Functioning (GAF), the WHO-5 scale, and medical resources used, which are part of a separate manuscript. The data collected in this study in a heterogeneous patient sample were intended to confirm the results of the previous efficacy studies [20, 21], which were conducted in a homogenous population of patients and which were intended for regulatory approval of AOM. Thus, we hypothesized that 6 months of AOM treatment would significantly improve psychopathology in patients receiving usual-care based AOM treatment.

## Methods

### Design

This multicenter, prospective, non-interventional study was designed according to the German Medicinal Products Act and approved by the Freiburg ethics commission international (Approval number: 014/1336). Diagnosis, treatment and monitoring of patients were conducted in a naturalistic, usual care treatment setting. Prospective collection of the data was chosen to collect high quality data, and a multicenter approach was chosen to ensure an adequate sample size. Patients were only recruited for the study after the treating psychiatrist had chosen AOM treatment for these patients based on clinical grounds. Since this was a naturalistic sample, combinations with other psychiatric medications, including oral antipsychotics were based on clinician’s choice. Participation in the study had no influence on treatment choice.

**Fig. 1:**
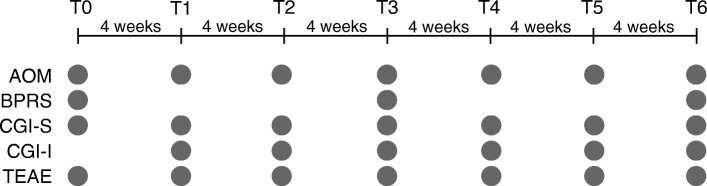
Study design. Patients were treated with AOM at seven time points (T0-T6) that were each 4 weeks apart. Data for the different endpoints were collected at the indicated time points. AOM, Aripiprazole once-monthly; BPRS, Brief Psychiatric Rating Scale; CGI-S, Clinical Global Impression – Severity; CGI-I, Clinical Global Impression – Improvement; TRAE, treatment related adverse events

Seventy-five centers participating in this study were located throughout Germany. The observation period for each patient was about 6 months. Data were collected from July 2014 to March 2016, when the last patient of the target sample had reached 6 months. Data were collected at seven time points (T0-T6), each about 4 weeks apart (− 2/+ 5 days). BPRS data were collected at T0, T3 (month 3) and T6 (month 6), and data for all other endpoints were collected at each monthly time point (except CGI-I, no baseline assessment possible; Fig. 1).

### Patients

Patients in the study cohort were ≥ 18 years old and diagnosed with schizophrenia (F20.X) according to ICD-10. Starting from the time of inclusion in the study, patients were treated with AOM on an outpatient basis according to prescribing information and clinical need. The treatment choice was independent from the study. All patients gave written informed consent.

Exclusion criteria for the study were contraindications for AOM, being a member or being related to a member of the study staff, pregnancy, planning a pregnancy, breastfeeding, or expected reluctance to follow the prespecified monitoring plan (as assessed by the treating psychiatrist).

### Assessments

The BPRS is a clinician-rated scale [23] which is well established in clinical routine, and therefore it was used as the primary endpoint in this study. Eighteen items were assessed that are grouped into 5 domains: anxiety/depression, anergia, thought disorders, activation and hostility/mistrust. Each of the respective items was rated on a seven-point scale ranging from 1 (not present) to 7 (extremely severe).

The Clinical Global Impression - Severity scale (CGI-S) was used to report the current severity of the patient’s mental illness on a seven-point scale ranging from 1 (normal, not at all ill) to 7 (extremely ill) [24]. This rating was complemented by the CGI-I (Improvement) scale, in which improvement of the mental illness from the beginning of the study is rated on a seven-point scale (1, very much improved to 7, very much worse).

Any patient reported adverse events were documented during the study and rated by the clinicians as treatment related adverse events (TRAE) or unrelated to treatment. Patients were asked about adverse events, comorbidities and comedications at every visit. Adverse events were coded according to MedDRA 19.0.

Other rating scales used in the same study will be discussed elsewhere.

### Statistical analysis

The primary outcome, change in BPRS total score from baseline to endpoint was analyzed using the Wilcoxon Signed-Rank test for paired samples, and the Wilcoxon’s rank-sum test for independent samples. Changes in marginal distributions in contingency tables of categorical outcomes were analyzed using Bhapkar’s test and proportions within one group of patients with the binomial test. Fisher’s exact test was used to compare proportions between groups of patients. Missing values were imputed using the Last Observation Carried Forward (LOCF) method if there was a value for T0 and at least one post-baseline time point. There was one prespecified subgroup analysis for the primary outcome, i.e., the comparison of patients aged ≤35 years old vs patients aged > 35 years old, as the younger age group had performed significantly better than the older age group in a prior AOM efficacy study [25]. Subgroup comparisons by age group were covaried using a linear regression analysis with backward selection of effects for variables that differed significantly between the two age groups at baseline at *p* < 0.05 (see Table 1). All data were processed using SAS™ software (SAS Institute, Cary, NC/USA), with all tests being two-sided and alpha = 0.05, without correction for multiple testing for secondary outcomes.

**Table 1:** Patient baseline demographics

Characteristic	Total (*n* = 242)	Patients aged ≤35 years (*n* = 89)	Patients aged > 35 years (*n* = 153)	*P*-value
Age, mean (SD), years	43.1 (15.1)	28.8 (4.3)	51.3 (12.7)	< 0.0001^a^
Sex, male, n (%)	133 (55.0)	57 (64.0)	76 (49.7)	0.0328^b^
Family status, n (%)				<. 0001^b^
Single	151 (62.7)	74 (84.1)	77 (50.3)	
Married/in a relationship	53 (22.0)	13 (14.8)	40 (26.1)	
Divorced	23 (9.5)	1 (1.1)	25 (16.3)	
Widowed	11 (4.6)	0 (0.0)	11 (7.2)	
Employment status, n (%)				<. 0001^b^
Employed	43 (18.0)	23 (26.4)	20 (13.2)	
Unemployed	73 (30.5)	36 (41.4)	37 (24.3)	
Annuitant	99 (41.4)	13 (14.9)	86 (56.6)	
Housewife/househusband	11 (4.6)	2 (2.3)	9 (5.9)	
In school/education/re-education	13 (5.4)	13 (15.0)	0 (0.0)	
Duration of untreated psychosis, mean (SD), years	1.2 (8.0)	1.4 (3.2)	1.0 (9.8)	0.7719^a^
Age at diagnosis, mean (SD), years	30.9 (13.0)	23.0 (4.3)	35.3 (14.2)	<. 0001^a^
Time of diagnosis, n (%)				<. 0001^b^
Within the last 5 years	78 (32.4)	47 (53.4)	31 (20.3)	
More than 5 years ago	163 (67.6)	41 (46.6)	122 (79.7)	
No. of illness episodes, n (%)				0.0002^b^
First episode of schizophrenia	19 (7.9)			
≤ 5 episodes	137 (57.1)	64 (72.7)	73 (48.0)	
> 5 episodes	103 (42.9)	24 (27.3)	79 (52.0)	
BMI, mean (SD), kg/m^2^	29.3 (6.9)	28.6 (7.3)	29.7 (6.6)	0.2381^a^
Underweight (<18,5), n (%)	3 (1.3)	3 (3.5)	0 (0.0)	
Normal weight (18,5 < 25), n (%)	59 (24.6)	21 (24.1)	38 (24.8)	
Overweight (25 < 30), n (%)	95 (39.6)	36 (41.4)	59 (38.6)	
Obese (≥ 30), n (%)	83 (34.6)	27 (31.0)	56 (36.6)	
BPRS at baseline, mean (SD)	54.1 (15.6)^c^	53.0 (16.1)^d^	53.7 (15.9)^d^	0.7608^a^
CGI-S at baseline, mean (SD)	4.8 (0.8)	4.7 (0.9)	4.8 (0.8)	
CGI-S at baseline, n (%)				0.7081^b^
Mildly ill	12 (5.0)	6 (6.8)	6 (4.0)	
Moderately ill	79 (32.9)	30 (34.1)	49 (32.2)	
Markedly ill	100 (41.7)	33 (37.5)	67 (44.1)	
Severely ill	48 (20.0)	19 (21.6)	29 (19.1)	
Extremely ill	1 (0.4)	0 (0.0)	1 (0.7)	

In cases where percentages do not add up to 100, data were missing for some patients

*BMI* body mass index, *BPRS* Brief Psychiatric Rating Scale, *CGI-S* Clinical Global Impressions-Severity Scale, *SD* standard deviation

^a^t-Test

^b^Fisher’s exact Test

^c^full analysis set (last observation carried forward)

^d^all values

## Results

### Baseline assessments

Two hundred seventy eight patients were reported by the treating clinicians to be eligible for the study. After screening, 243 patients were included in the study. Patient baseline demographics are presented in Table 1. Altogether, 204 patients (84.3%) completed all scheduled visits, and 23 patients (9.5%) came for at least the first and last visits.

Diagnoses included paranoid schizophrenia (ICD-10: F20.0) in 202 patients (83.5%), non-differentiated schizophrenia in 22 patients (9.1%; ICD-10: F20.3) and others for the remaining 18 patients (7.4%). All patients had been previously treated with oral aripiprazole for a mean duration of 9.7 months (SD 22.3 months), with 33.3% of patients having received oral aripiprazole for < 1 month, 39.9% for 1–6 months, and 26.8% for more > 6 months. Apart from aripiprazole treatment, 141 patients (58.3%) had been treated with additional medication, and the most common substances were risperidone, quetiapine, and olanzapine (35, 34 and 28 patients, respectively, being 14.5%, 14,1, and 11.6% of the patient population).

At study start, 20 patients (8.5%) received 5 mg oral aripiprazole, 183 (77.5%) received 10–20 mg, and 33 (14.0%) received a higher oral aripiprazole dose. During oral aripiprazole treatment, most patients had been symptomatically stable, as assessed by the treating psychiatrist (without use of a dedicated rating scale), for a mean duration of 5.9 months (standard deviation 18.2), with 91 patients (39.2%) being stable for < 1 month and 28 (12.1%) being not stable at all. At the start of the study 81 patients (33.5%) received further medication apart from aripiprazole (Table 2).

**Table 2:** Comorbidities and comedications

Frequent comorbidities, n (%)	
Hypertension	24 (9.9)
Depression	20 (8.3)
Diabetes mellitus	16 (6.6)
Obesity	13 (5.4)
Hyperthyroidism	7 (2.9)
Anxiety	5 (2.1)
Frequent comedications for treatment of somatic diseases, n (%)	
Metformin	13 (5.4)
Bisoprolol	12 (5.0)
Ramipril	10 (4.1)
Frequent comedications for treatment of mental diseases, n (%)	
Venlafaxine	15 (6.2)
Duloxetine	6 (2.5)
Mirtazapine	5 (2.1)
Benzodiazepines	70 (28.9)
Additional drugs to treat schizophrenia at the start of the study, n (%)	
Quetiapine	21 (8.7)
Olanzapine	13 (5.4)
Clozapine	12 (5.0)

Reasons for the decision to switch from treatment with oral aripiprazole to AOM were most often (48.4%) easier adherence to treatment, followed by good/better tolerability (16.9%), patient’s request (13.6%), good/better efficacy (12.0%) and easier use (9.1%) than oral treatment. Switching was a subjective decision of treating clinicians to further improve outcome in a naturalistic sample of patients. At study start, 79.3% of patients received 400 mg of injectable aripiprazole, 17.4% received 300 mg, 2.9% received 200 mg and one patient (0.4%) received 160 mg. Most patients (132, 54.6%) were treated with 400 mg of injectable aripiprazole at every time point T0-T6, and 12 patients (5.0%) received 300 mg at all time points. Of the remaining patients, 30.6% were treated with different AOM doses, and 9.9% discontinued treatment. Reasons for discontinuation of AOM included patient’s request (9, 3.7%), lack of effectiveness (7, 2.9%), adverse drug reactions (6, 2.5%), and patients not arriving at agreed appointments, having moved to a new house, or undergoing inpatient drug addiction therapy (1 case each, 0.4% each; one patient gave two reasons for discontinuation). AOM adherence was monitored based on injection visits taking place, yielding that 84% of the patients were fully adherent, 8% were adherent to receiving > 70% of injections, and that 8% had lower adherence rates. After the 6-month observational period, 200 patients (89.3%) decided to continue AOM treatment.

**Fig. 2:**
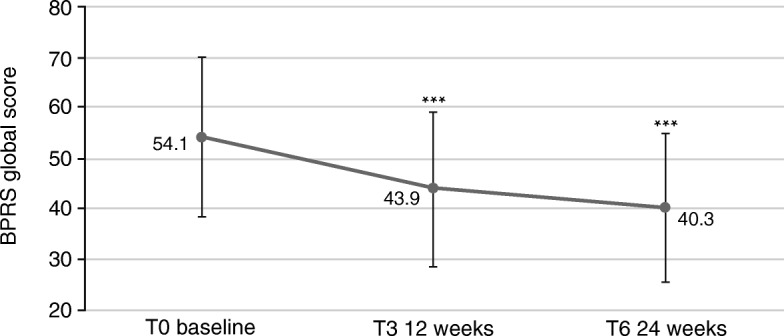
Effects of AOM treatment on Brief Psychiatric Rating Scale (BPRS), global score (sum of values for all items). 18: symptoms not present, 126: symptoms extremely severe. *** *p* < 0.001. Error bars represent standard deviations

At baseline, the CGI-S category with the most patients (41.7%) was “markedly ill”. The mean global BPRS value was 54.1 (±15.6, standard deviation, SD) (Fig. 2), and 94.5% presented with a GCI-S value of ≥4 (moderately severe) (Fig. 5).

### Brief psychiatric rating scale (BPRS)

**Fig. 3:**
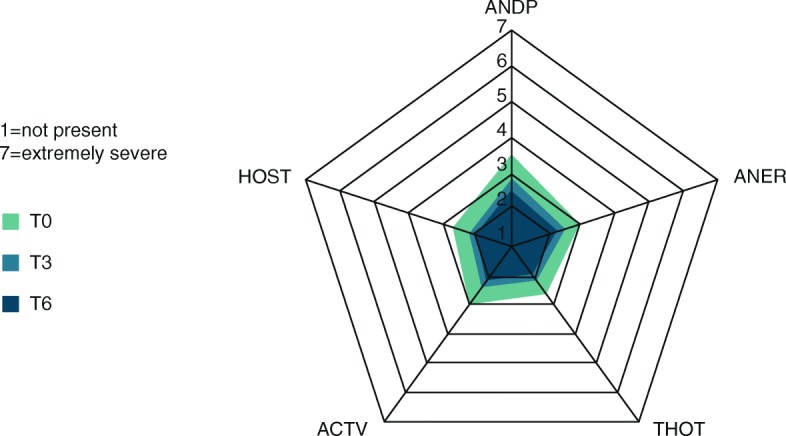
Profile for each score at baseline (T0), follow-up visit (T3) and last visit (T6) assessed by BPRS. ANDP: anxiety/depression; ANER: anergia; THOT: thought disorders; ACTV: activation; HOST: hostility/mistrust

The psychopathological status of the patients improved significantly during treatment, as assessed via the BPRS (*p* < 0.0001, Fig. 2). One hundred eighty-seven patients (82.0%) improved during the study, 12 (5.3%) remained the same, and 29 (12.7%) had worse global BPRS scores at T6, seven of which worsened by more than 10 points. Altogether, 131 patients (57.5%) had a reduction of their global BPRS value by at least 20%. In total, the difference in global BPRS between T6 and T0 was − 13.8 (SD: 16.0; 95% CI: [− 15.9; − 11.7]; *p* < 0.001). The largest improvements were found in the scores anxiety/depression (T6-T0: -0.98; SD: 1.21; 95% CI: [− 1.14; − 0.83]) and activation (T6-T0: -0.82; SD: 1.15; 95% CI: [− 0.97; − 0.67]) (Fig. 3; score reduction must be multiplied by number of respective items to obtain total score reduction).

**Fig. 4:**
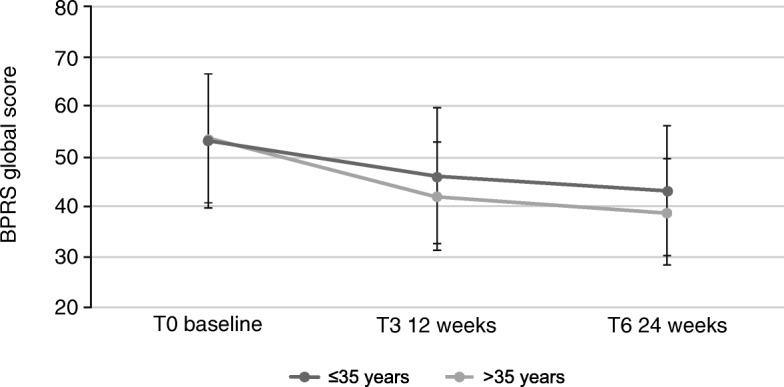
Effects of AOM treatment on Brief Psychiatric Rating Scale (BPRS), global score (values of all items added up) in patients ≤35 years or > 35 years. 18: symptoms not present, 126: symptoms extremely severe. Error bars represent standard deviations

For patients ≤35 years, the difference between global BPRS values at T6 and T0 was − 17.2 (SD: 17.6; 95% CI: [− 21.1; − 13.4]). For patients > 35 years, the difference was − 11.9 (SD: 14.6; 95% CI: [− 14.3; − 9.5]) (Fig. 4). This difference was not significant (*p* = 0.0746, Wilcoxon two-sample test). The differences in subscores are presented in Table 3.

**Table 3:** Differences in BPRS subscores, stratified by patient age

BPRS subscore	≤35 years T6-T0 (SD; [95% CI])	> 35 years T6-T0 (SD; [95% CI])
Anxiety/depression	−1.19 (1.21; [−1.46; −0.93])	−0.87 (1.20; [− 1.06; − 0.67])
Anergia	− 0.83 (1.08; [− 1.06; − 0.59])	−0.57 (0.83; [− 0.71; − 0.43])
Thought disorders	−0.92 (1.08; [− 1.15; − 0.68])	−0.54 (0.86; [− 0.69; − 0.40])
Activation	−0.97 (1.19; [− 1.23; − 0.71])	−0.74 (1.13; [− 0.92; − 0.55])
Hostility/mistrust	−0.85 (1.23; [− 1.12; − 0.58])	−0.57 (0.98; [− 0.74; − 0.41])

All differences were significant (*p* < 0.001)

*BPRS* Brief Psychiatric Rating Scale

### Clinical global impression (CGI)

**Fig. 5:**
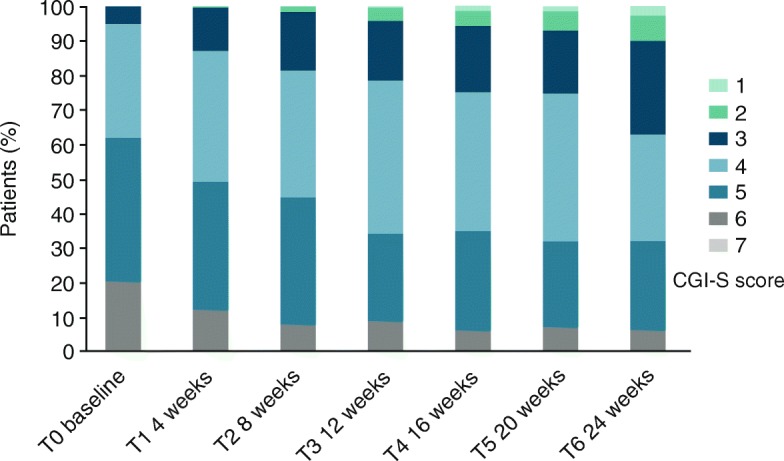
Clinical Global Impression – Severity (CGI-S), scores between 1 (not at all ill) and 7 (among the most extremely ill patients). Percentage of patients with each score is shown

Severity of illness as the secondary study outcome was measured using the CGI-S score. During the study, the proportion of patients with high scores became smaller, while the proportion with low scores increased (Fig. 5). At baseline, 146 patients (62.1%) had a severity score of 5 (markedly ill) or worse. At T6, only 75 (31.9%) remained with a score of 5 or 6. By contrast, at baseline only 12 patients (5.1%) scored as “mildly ill” (3), whereas at T6, 87 patients (37.0%) had a score of 3 or better. These improvements were found to be highly significant (*p* < 0.001) using Bhapkar’s test.

**Fig. 6:**
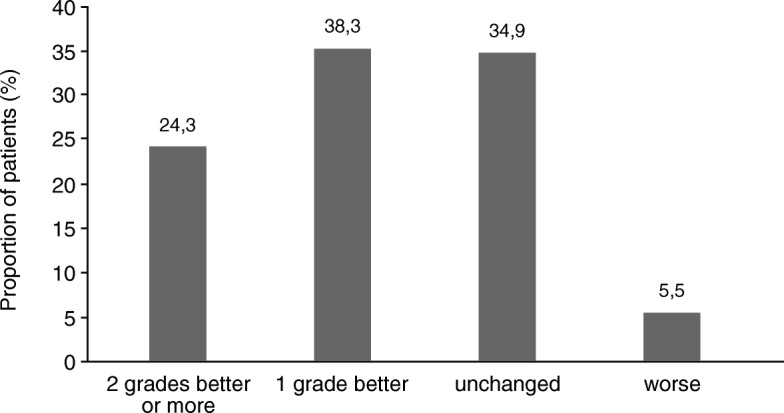
Percentages of patients with defined changes in the CGI-S score between T0 and T6. Data were missing for 7 patients

According to the CGI-S scores, 222 patients (94.5%) were stable (scores improved or remained the same) during the study. Altogether, 57 patients (24.3%) improved by two points or more (Fig. 6). Thirteen patients (5.4%) deteriorated, with a mean score of 1.3 points on the scale (four patients deteriorated by two points, another nine patients by one point each).

These improvements were also reflected by CGI-I scores: At T6, 35 (15.2%) of patients were rated as very much improved (score of 1) compared to baseline, and 96 (42.0%) were rated as much improved (score of 2).

In patients > 35 years, 18.8% (*n* = 28) improved by 2 points or more on the CGI-S scale, whereas in younger patients (≤35 years), this was true for 33.7% (*n* = 29) of patients. Only 8.8% (*n* = 13) of older patients had a CGI-I score of 1 (very much improved) at T6, whereas in younger patients, this was the case for 26.2% (*n* = 22). The difference between age groups regarding CGI-I scores at T6 was significant (*p* = 0.0074, Fisher’s exact test).

### Adverse events

During the study, a total of 153 adverse events (AE) were reported by patients (Table 4) and coded according to MedDRA 19.0. One hundred thirty-three of these AEs were rated by the clinician as probably or possibly treatment-related (TRAE). Only one type of event, “medication taken at an inappropriate time”, applied to more than 5% of patients (24.8%). In most of these cases, oral aripiprazole had been discontinued earlier than recommended (< 2 weeks of the recommended concomitant treatment after first injection). Altogether, 52 TRAEs (39.1%) improved during the treatment period. Extrapyramidal symptoms and weight disturbances that were reported as treatment-related were rare (Table 4). No TRAEs were recorded related to sexual dysfunction, such as hyperprolactinemia. No patients died during the study.

**Table 4:** Adverse Events (AE), coded according to MedDRA19.0

Adverse Events, n (% of patients)	
Any AE	153 (42.2)
TRAE	133 (39.7)
Specific TRAE occurring in ≥5% of patients	
Medication taken at inappropriate time^a^	60 (24.8)
TRAEs related to extrapyramidal symptoms	7 (2.9)
Akathisia	1 (0.4)
Dystonia	0 (0)
Extrapyramidal disorder	1 (0.4)
Muscle rigidity	0 (0)
Muscle spasms	1 (0.4)
Tremor	2 (0.8)
Parkinsonism	1 (0.4)
Parkinson gait	1 (0.4)
TRAEs related to weight change	1 (0.4)
Weight increased	1 (0.4)
Weight decreased	0 (0)

Treatment related adverse events (TRAE) were defined as events probably or possibly caused by the medication as estimated by the treating clinician

^a^In most cases, oral aripiprazole was discontinued at an earlier time than recommended

## Discussion

The therapeutic effect of AOM treatment in schizophrenia was shown in previous RCTs, which were initiated to determine its efficacy [20, 21, 25]. Furthermore, the effect of oral aripiprazole was studied in short-term as well as long-term therapy for bipolar disorder [26]. AOM has also been approved by the FDA for the treatment of bipolar disorder [27] and a real-world study has been conducted on AOM use for both bipolar disorder and schizophrenia [28]. However, in Europe, AOM is only approved for schizophrenia treatment. Here, we report prospectively collected real-world data in a relatively large cohort in Germany. This prospective non-interventional study was conducted to support and amplify the effectiveness of AOM treatment in a naturalistic setting of usual-care patients being treated with oral aripiprazole before enrollment in the study.

The main findings were that during the 6-month observation period, patients experienced significant improvements in psychopathology and in severity of illness scale scores. The global BPRS values were significantly better already after 12 weeks (*p* < 0.001), with further significant improvement after 24 weeks (*p* < 0.001). This effect was numerically more prominent in younger patients ≤35 years.

In line with the results from the BPRS, CGI-S and CGI-I values also improved significantly during the study period. There were fewer patients with high scores and more patients with low scores at the end of the study, with significant differences between start and end of the study. This finding is in agreement with the improvements seen in the CGI-S values of patients in a previous study [25]. The improvements were significantly stronger in younger patients ≤35 years.

The results of numerically or statistically significant greater improvements in the younger age group further highlight the importance of early and consequent therapeutic intervention, which may help protect patients from the potential deteriorating effects of experiencing repeated psychotic episodes [29–31].

Some patients worsened during the study. Altogether, 29 patients (12%) achieved worse ratings on the BPRS, and 13 patients (5.4%) were considered worse on the CGI-S scale. In most cases, the deterioration was minimal and may have been the result of normal variation of the patient’s status. Seven patients worsened by 10 points on the BPRS and four patients worsened by two points on the CGI-S. However, other studies found similar rates of deterioration among their patients. [21] In the registration trial for AOM by Kane et al., 10% of patients in the active group experienced exacerbation of psychotic symptoms or impending relapse despite receiving ongoing AOM treatment [20]. In the European registration trial, Kaplan–Meier estimated impending relapse rates at week 26 from randomization were 7.1% for AOM 400 mg [21]. Finally, the proportion of patients on AOM or oral aripiprazole meeting exacerbation of psychotic symptoms/relapse criteria at the endpoint of a study by Ishigooka et al. was 6.6% [32]. Reasons for breakthrough psychosis may be psychosocial, comorbidity-related, pharmacokinetic/administration-related, or biological in nature [33], yet the exact reasons for the worsening in the patients in this study are unknown to us.

Among other variables, adherence to psychopharmacological treatment is considerably influenced by the occurrence of side effects of the medication [34]. Particularly side effects, such as weight gain, experiencing extrapyramidal symptoms, or side effects related to hyperprolactinemia, such as sexual dysfunction, are common causes for stopping medication [35]. In our study, these side effects were rare. According to our medication monitoring, 84% of the patients were fully adherent to medication, which supports the notion, that patients prefer an effective and well tolerable psychopharmacological treatment.

Interestingly, only few TRAEs were recorded during the study, which were not different in the age subgroup analyses. TRAEs related to extrapyramidal symptoms (2.9%), weight increase (0.4%) or hyperprolactinemia (0%) were very rare. Extrapyramidal symptoms were only found in patients > 35 years who were diagnosed with schizophrenia > 5 years ago.

Although LAIs can lead to improved adherence rates and potentially to improved long-term outcomes, there has been some debate about the effectiveness of LAIs in the long-term treatment of schizophrenia: the advantages of LAIs in comparison to continuous oral medication have been repeatedly questioned. However, comparison of different study types, such as RCTs [36], mirror image studies [12], and retrospective as well as prospective cohort studies [14] revealed that differences regarding the effectiveness of LAIs are likely dependent on how representative and severely ill the studies sample is [37, 38].

Studies suggested that therapeutic alliance is one of the most important factors affecting adherence [34]. Moreover, studies of collaborative care, which can increase the alliance between the treatment team and the patient, and which respects the patients and their autonomy, have led to significant improvements in adherence rates. We believe that focusing on therapeutic alliance via provision of concurrent psychosocial interventions is important to optimize outcomes, even while using long-acting injectable antipsychotics [39, 40].

In contrast to RCTs with a highly selected study population, both naturalistic study types in general showed better effectiveness. In our study, we could broaden and support findings from RCTs with AOM and show that comparable efficacy results could be obtained.

The observed statistically and clinically relevant improvements in psychopathology and severity of illness ratings are particularly noteworthy, as patients had already received an average of 9.7 months of oral aripiprazole, and 87.9% were deemed by the clinicians as “clinically stable” and for an average of 5.9 months. Thus, the additional improvements in a real-world sample are at least in some relevant part attributable to a change in formulation of the antipsychotic and the related more consistent and reliable delivery of the antipsychotic medication [13]. The fact that 89.3% of the patients decided to continue with AOM after the 6-month observational period further underscores the acceptability of this treatment option.

These findings are in line with the study comparing aripiprazole and paliperidone LAI by Naber et al. [25] where the CGI-S scores also improved continuously during treatment with AOM. In the RCT studies of AOM compared to placebo by [20, 32] the PANSS score remained stable for 52 weeks, and in the noninferiority study compared to oral aripiprazole by Fleischhacker et al., [41] the PANSS score was stable for 38 weeks after switching to treatment with AOM. Similarly, a study comparing risperidone LAI with oral antipsychotics for 1 year found that frequent medication switches led to less favorable outcomes [42], suggesting that antipsychotic medication stability might be an important factor for improved or stable outcomes in patients with schizophrenia.

Similar positive results for AOM treatment as in our study were found in another naturalistic study that used the Positive and Negative Syndrome Scale (PANSS) and CGI scales as endpoints [43]. In that study, highly significant improvements were found as early as 4 weeks after study start.

Finally, in the past, observational studies on the use of aripiprazole in patients with bipolar disorder also confirmed the efficacy results of RCTs [44], showing that aripiprazole was effective both in controlled experimental settings and in real world clinical practice settings.

Due to its naturalistic, non-interventional design, this study has several limitations. Importantly, there is a risk of a selection bias (e.g., patients were willing to take LAI medication), and of an expectation bias (knowledge of being on the LAI formulation of aripiprazole and of being in a study), there are no reference points other than baseline (patient’s condition at T0), and due to a lack of any randomization there are possible confounding factors, which cannot be identified or excluded. The dose of AOM varied during the study period, which can also be a confounding factor. Finally, all patients had already received and tolerated oral aripiprazole. Nevertheless, the initiation with oral aripiprazole before starting AOM is according to the package insert and clinical guidelines, and inclusion of patients who were selected under usual care conditions as being clinically eligible for AOM treatment increases the external validity and generalizability of the findings.

In general, most often, observational studies produce results that are similar to those of RCTs [45–47], although in some cases the magnitude of an observed effect is different [48–50]. If the observational studies are well-designed, their results do not seem to systematically overestimate treatment effects from RCTs [51, 52]. Therefore, observational studies are an important complement to RCTs [53] in order to evaluate the feasibility of a given treatment and generalizability of the findings to less restricted patient populations, both regarding the magnitude of efficacy and of safety signals.

## Conclusions

Taken together, our results support previous results of the efficacy, tolerability and acceptability of AOM under routine clinical practice conditions. Outpatients who already respond to oral aripiprazole can further benefit from treatment with AOM, which proved to be effective under routine clinical conditions in a “real-life” sample of patients with schizophrenia. TRAEs were rare, a finding that is particularly important for patients who often undergo long-term antipsychotic treatment and who frequently have psychiatric and medical comorbidities and receive comedications, each of which are restricted in RCTs. The positive therapeutic effect was pronounced in younger patients, which underlines the need for early and continuous treatment.

## Abbreviations

AE: Adverse event
AOM: Aripiprazole once-monthly
BPRS: Brief psychiatric rating scale
CGI-I: Clinical global impression – improvement
CGI-S: Clinical global impression – severity
ICD: International classification of diseases
LAI: Long-acting injectable
LOCF: Last observation carried forward
PANSS: Positive and negative syndrome scale
RCT: Randomized controlled trial
SD: Standard deviation
TRAE: Treatment related adverse event
