# Correction to: Effectiveness of aripiprazole once-monthly in schizophrenia patients pretreated with oral aripiprazole: a 6-month, real-life non-interventional study

**DOI:** 10.1186/s12888-018-1968-4

**Published:** 2018-12-14

**Authors:** Daniel Schöttle, Wolfgang Janetzky, Daniel Luedecke, Elmar Beck, Christoph U. Correll, Klaus Wiedemann

**Affiliations:** 1 Klinik für Psychiatrie und Psychotherapie, Zentrum für Psychosoziale, Medizin, Universitätsklinikum Hamburg-Eppendorf, Martinistrasse 52, 20, ,246 Hamburg, Germany; 2 Lundbeck GmbH, Ericusspitze 2, 20, ,457 Hamburg, Germany; 3 ANFOMED GmbH, Röttenbacher Str. 17, 91,096, Möhrendorf, Germany; 4 Department of Psychiatry, The Zucker Hillside Hospital, Northwell Health, 75–59 263rd St, Glen Oaks, NY 11004 USA; 5 Department of Psychiatry and Molecular Medicine, Hofstra Northwell School of Medicine, 500 Hofstra Blvd, Hempstead, NY 11549 USA; 6 Department of Child and Adolescent Psychiatry, Charité Universitätsmedizin, Augustenburger Platz 1 (Mittelallee 5A), 13, ,353 Berlin, Germany

**Correction to: BMC Psychiatry (2018) 18:365**

**https://doi.org/10.1186/s12888-018-1946-x**

Following publication of the original article [1], the authors notified us that the color labelling in Fig. 4 was incorrect.

The corrected Fig. 4 is presented below.

**Fig. 4:**
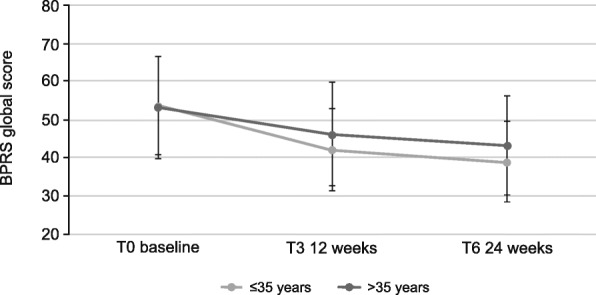
Effects of AOM treatment on Brief Psychiatric Rating Scale (BPRS), global score (values of all items added up) in patients ≤35 years or > 35 years. 18: symptoms not present, 126: symptoms extremely severe. Error bars represent standard deviations
